# First genome-wide CNV mapping in *FELIS CATUS* using next generation sequencing data

**DOI:** 10.1186/s12864-018-5297-2

**Published:** 2018-12-10

**Authors:** F. Genova, M. Longeri, L. A. Lyons, A. Bagnato, the 99Lives Consortium, Barbara Gandolfi, Danielle Aberdein, Paulo C. Alves, Gregory S. Barsh, Holly C. Beale, Tomas F. Bergström, Adam R. Boyko, Jeffrey A. Brockman, Marta G. Castelhano, Patricia P. Chan, Brian W. Davis, Ottmar Distl, Nicholas H. Dodman, N. Matthew Ellinwood, Jonathan E. Fogle, Oliver P. Forman, Dorian J. Garrick, Jens Häggström, Christopher R. Helps, Marjo K. Hytönen, Daniel M. Ibrahim, Maria Kaukonen, Christopher B. Kaelin, Emilie Leclerc, Teri L. Lear, Tosso Leeb, Hannes Lohi, Darío G. Lupiáñez, Mark A. Magnuson, Richard Malik, Michael J. Montague, John S. Munday, William J. Murphy, Elaine A. Ostrander, Niels C. Pedersen, Simon M. Petersen-Jones, Max F. Rothschild, Beth Shapiro, Joshua A. Stern, William F. Swanson, Karen A. Terio, Rory J. Todhunter, Yu Ueda, Wesley C. Warren, Elizabeth A. Wilcox, Julia H. Wildschutte, Edward I. Ginns, M. G. Strillacci

**Affiliations:** 1 Department of Veterinary Medicine, University of Milan, 20122 Milan, Italy; 2 Department of Veterinary Medicine and Surgery, College of Veterinary Medicine, University of Missouri, Columbia, MO 65211 USA

**Keywords:** CNV, CNVR, Cn.MOPS, CNVnator, NGS, *Felis catus*, Cat breeds

## Abstract

**Background:**

Copy Number Variations (CNVs) have becoming very significant variants, representing a major source of genomic variation. CNVs involvement in phenotypic expression and different diseases has been widely demonstrated in humans as well as in many domestic animals. However, genome wide investigation on these structural variations is still missing in *Felis catus.* The present work is the first CNV mapping from a large data set of Next Generation Sequencing (NGS) data in the domestic cat, performed within the 99 Lives Consortium.

**Results:**

Reads have been mapped on the reference assembly_6.2 by Maverix Biomics. CNV detection with cn.MOPS and CNVnator detected 592 CNVs. These CNVs were used to obtain 154 CNV Regions (CNVRs) with BedTools, including 62 singletons. CNVRs covered 0.26% of the total cat genome with 129 losses, 19 gains and 6 complexes. Cluster Analysis and Principal Component Analysis of the detected CNVRs showed that breeds tend to cluster together as well as cats sharing the same geographical origins. The 46 genes identified within the CNVRs were annotated.

**Conclusion:**

This study has improved the genomic characterization of 14 cat breeds and has provided CNVs information that can be used for studies of traits in cats. It can be considered a sound starting point for genomic CNVs identification in this species.

**Electronic supplementary material:**

The online version of this article (10.1186/s12864-018-5297-2) contains supplementary material, which is available to authorized users.

## Background

Short Tandem Repeats and Single Nucleotide Variants (Single Nucleotide Polymorphism – SNPs, as they are known) have been widely used in the study of the genome diversity and inherited diseases for a long time. Other structural and more complex variants - like Copy Number Variants (CNV) - were also known [1], and the recent advances in genome technologies, especially the development of array platforms and next generation sequencing, has allowed more global analyses of CNVs at a genome-wide level. These variants consist of changes in copy number of DNA sequences in comparison to a reference genome, including duplications (gain state) and deletions (loss state). The absence of variations is defined as normal state [2]. Conventionally, CNVs are defined as 1 Kb to several Mb in length, therefore being distinct from the smaller structural variants as indels and are more variable than SNPs, which vary by a single base pair [3].

An early study of the completed human genome for large-scale copy-number variants (LCVs) identified approximately 200 polymorphisms within the genomes of 20 normal individuals [4]. In another study, the regions including LCVs overlapped with genes involved in human disease syndromes and cancer, such as *CMT4B2* gene (Charcot-Marie-Tooth disease type 4B2) and *DPY*, *LRP12*, *FOG2* genes (squamous cell carcinoma) [5]. CNVs not only influence human phenotypes but are also related to genome evolution. The location and frequencies of the human and chimpanzee CNVs have been compared and duplications and deletions of genes with functions related to cell proliferation and inflammatory response have been found. In particular, a loss of the chimpanzee *TBC1D3* gene, compared to the eight paralog copies of this gene in the human reference individual, may reflect a positive selection and adaptive phenotypic differentiation during primate evolution [6]. These studies used array-based comparative genomic hybridization methods (array CGH, aCGH), which lacked sensitivity to detect a wide range of CNVs [7].

The introduction of SNP high density genotyping and Next Generation Sequencing (NGS) approaches, together with the development of new bioinformatics tools, has led to better strategies for CNV detection [8, 9]. The 1000 Genomes Project Consortium (2010), using 179 low coverage whole genome human sequences, identified 28,025 structural variants, suggesting that CNVs represent 4.8–9.5% of the human genome [9]. NGS is also an alternative tool for genotyping CNVs associated to diseases, such as inherited kidney diseases [10].

Genome-wide studies to detect CNVs have been extended to domestic animals. CNVs have been shown to affect phenotypes such as pigmentation, morphology and production traits [11, 12]. CNVs have also been suggested to be responsible for complex disease traits such as periodic fever syndrome in Shar-Pei dogs [13] and canine squamous cell carcinoma of the digit [14].

NGS has supported the discovery of CNVs in animals too, providing higher sensitivity and allowing their identification at a genome-wide level in cattle [15], chickens [16], mice [17] and dogs [18]. Recently, CNV detection with NGS data has been used for association studies on production traits in livestock, such as fatty acids dynamics in beef cattle [19].

The genome assembly of the domestic cat is based on approximately 3× Sanger sequencing and ~ 14× short-read Illumina-based NGS. This assembly_6.2 and the re-sequencing of additional breed individuals have identified a wealth of genetic variation within the cat genome which led in turn to the development of a successful DNA 63 K array [20]. However, an analysis of large structural variants as CNV in the domestic cat across breeds and individuals has not been attempted. New cat genomes are now available from the 99 Lives cat genome sequencing project, which has produced high quality NGS data of 30× coverage, using similar techniques and technologies [21–24]. In such a context, the present study is the first genome-wide CNV detection in the domestic cat and is based on the sequence data from the cat 99 Lives Project. Ultimately this work is meant to assist the evaluation of cat breeds and to be used for the association of CNVs to breed-specific phenotypes, including disease phenotypes.

## Materials and methods

### Samples

Forty-two whole genome sequences representing 14 different cat breeds were available from the 99 Lives Cat Genome Sequencing Initiative (http://felinegenetics.missouri.edu/99lives) [21]. The genome sequences were produced as previously described [22] and sequencing data are available at NCBI BioProject PRJNA308208. The represented breeds, the number of individuals per breed and additional sequencing information are reported in Table 1.

**Table 1:** List of the main cat breeds used in the study and number of individuals per breed

Breed	Sample/Breed	Library	Run Accession	Location
ABYSSINIAN (4)	ABY_1	~ 350 bp	SRR5373742	USA
	ABY_3		SRR6997541	Italy
	ABY_4	~ 350 bp	Under release	USA
BENGAL (2)	BEN_1	~ 550 bp	SRR5366704	USA
	BEN_2		Under release	Switzerland
BIRMAN (6)	BIR_1		SRR5055405	Asia
	BIR_2	~ 350 bp	Under release	USA
	BIR_3		Under release	Sweden
	BIR_4		Under release	Sweden
	BIR_5		Under release	Sweden
	BIR_6		Under release	Sweden
BRITISH SHORT HAIR (2)	BSH_1	~ 550 bp	SRR5358834	Western
	BSH_2	~ 550 bp	SRR5358833	Western
BURMESE (5)	BUR_1	~ 550 bp	SRR5373736	Western
	BUR_2	~ 550 bp	SRR5055402	Asia
	BUR_3	~ 550 bp	SRR5055400	Asia
	BUR_4	~ 350 bp	Under release	USA
	BUR_5	~ 350 bp	Under release	USA
DEVON REX (2)	REX_1	~ 550 bp	SRR5373726	Western
	REX_2	~ 550 bp	SRR5373735	Western
EGYPTIAN (1)	EGY_1	~ 350 bp	Under release	USA
MAINE COON (2)	MCO_1		Under release	Switzerland
	MCO_2		Under release	USA
NAPOLEON (3)	NAP_1	~ 550 bp	SRR5373738	Western
	NAP_2	~ 550 bp	SRR5373737	Western
	NAP_3	~ 550 bp	SRR5373734	Western
ORIENTAL SHORT HAIR (8)	OSH_1	~ 550 bp	SRR5358555	Asia
	OSH_2	~ 550 bp	SRR5358556	Asia
	OSH_3	~ 550 bp	SRR5358554	Asia
	OSH_4	~ 550 bp	SRR5358559	Asia
	OSH_5	~ 550 bp	SRR5358558	Asia
	OSH_6	~ 350 bp	Under release	USA
	OSH_7	~ 350 bp	Under release	USA
	OSH_8	~ 550 bp	SRR5358557	Asia
PERSIAN (1)	PER_1		SRR5055403	Western
RAGDOLL (2)	RAG_1		SRR5055399	Western
	RAG_2		SRR5055396	Western
SPHYNX (1)	SPH_1	~ 350 bp	Under release	USA
SIAMESE (4)	SIA_1		Under release	Asia
	SIA_2	~ 550 bp	SRR5363128	Asia
	SIA_3	~ 550 bp	SRR5363127	Asia
	SIA_4	~ 550 bp	SRR5363129	Asia

The genome data for all the cats were produced by Illumina-based short-read technology using PCR-free libraries of ~ 350 bp and/or ~ 550 bp. Most genomes are at ~30× coverage, with 100–150 bp paired end reads. All the reads were mapped to the cat reference assembly_6.2 by Maverix Biomics (http://www.ncbi.nlm.nih.gov/assembly/320798) [25].

### CNVs detection

Two programs using Read Depth (RD) based methods, cn.MOPS [26] and CNVnator [27], were employed to detect CNVs in the cat genomic data. Cn.MOPS and CNVnator software support the readily available bam files for the analysis and are considered the most suitable tools for Illumina sequencing data [28].

#### Cn.MOPS

The CNVs were firstly identified using the R “cn.MOPS” routine. The output was filtered to exclude false calls following the software manual indications, so only losses with median in the expected log fold change < − 1 and gains with median > 0.6 were considered, as described in detailes by the authors [26]. Window Length (WL) was set at 5 Kb.

#### CNVnator

The second CNV detection method utilized CNVnator. The filtering was carried out considering only CNVs with size ≥1 Kb, zero mapping quality (q_0_) < 0.5 and P_val_1 < 0.001. Since the sequences used for the analysis had ~20× - 30× coverage, the WL was set at 100 bp as suggested by the authors [27].

#### Consensus mapping of CNV, CNVR and data analysis

To reduce the false positive calls from each of the detection method, the CNVs obtained from cn.MOPS and CNVnator were compared using the -intersectBed command of Bedtools software [29]. Only CNVs that overlap for at least 80% (consensus_CNVs) were considered true calls and included in further analyses. When a different loss/gain state was identified by the two software for a specific CNV, a visual inspection of the sequence read depth was carried out for that CNV to identify the true state. Particularly, samtool -view option (http://samtools.sourceforge.net/) was used to extract the CNV sequence, including 50 K bp of the flanking regions, and the read depth was visually inspected with the Golden Helix GenomeBrowse. This allowed to identify false and true positive calls and assign the true CNVs state.

CNV regions (CNVRs) for all the cats were obtained by merging consensus_CNVs using the -mergeBed command of Bedtools [29]. In order to validate the CNVRs identified and to exclude any possible false positive call, all the identified CNVRs were validated by the visual inspection. The sequence read depths of all the cats pertaining to a CNVR, and thus showing a CNV in that region, were compared to the ones of two cats showing CNV normal state for that region. Only losses or gains with clear boundaries were considered true CNV calls, contributing to CNVRs. All singleton CNV calls were also visually validated to exclude false positive calls and then considered as CNVRs. All the CNVRs boundaries were validated and re-assigned with visual inspection: for both sides, the initial descending bp and the initial ascending bp positions were identified as boundaries for losses and gains respectively.

Genes within the CNVRs were identified using the annotation of the NCBI *Felis catus* assembly_6.2 gene dataset and the Bedtools -intersectBed command was used to catalogue genes in the corresponding regions. Gene Ontology Terms (GO) and Kyoto Encyclopedia of Genes and Genomes (KEGG) pathway analyses were performed with the DAVID Bioinformatic Database (https://david.ncifcrf.gov/tools.jsp). GeneCards database (www.genecards.org) was consulted to obtain information on the function of the identified genes. Imprinted Gene database (www.geneimprint.com) was used to identify imprinted genes among the ones located within the identified CNVRs.

#### Cat population analyses

Structure and genomic diversity among all the cats were examined using Principal Component Analysis (PCA) and a clustering analysis, grouping the individuals according to their CNVR similarities [30]. A scoring matrix of the CNVRs was developed by encoding a value of ‘0’ or ‘1’ according to the presence or absence of any mapped CNV in the corresponding CNVR. The matrix considered only the 92 CNVRs shared by two or more individuals. A hierarchical agglomerative clustering, based on Unweighted Pair-Group Average method (UPGMA), was applied to the scoring matrix using the *pvclust* function from the *pvclust* R package [31]. Multiscale bootstrap resampling was performed to calculate the Approximately Unbiased *P*-value (AU-P) using 10,000 bootstraps to assess the robustness of branches. The AU-P and Bootstrap Probability value (BP-P) are presented for each node, as well as the edge numbers. The PCA was performed with Past3 software using the same matrix of the clustering analysis [32].

## Results

### CNVs detection with cn.MOPS

After filtering, 2282 CNVs were identified using cn.MOPS software. A mean of 23 CNVs/cat was observed. The Siamese cat sample SIA_4 showed 585 CNVs and was excluded as an outlier. Therefore, 1697 CNVs were considered for downstream analyses.

### CNVs detection with CNVnator

After editing, CNVnator detected 285,533 CNVs. A mean of 5827 CNVs/cat was reported and the Siamese cat sample SIA_4 was also confirmed as an outlier. The Siamese cat sample SIA_2 was the cat with the highest number of CNVs (9413) while 4405 CNVs were counted in the Birman cat sample BIR_2, which was the one with the lowest number of CNVs. After the SIA_2 outlier exclusion, 234,484 CNVs were considered in downstream analyses.

### Consensus mapping of CNV, CNVR and data analysis

Comparing cn.MOPS and CNVnator detections, a total of 999 consensus_CNVs was obtained, representing 59% of the 1697 CNVs identified by cn.MOPS. Out of the 147 consensus_CNVs with different state, 78 were identified by visual inspection as true call and their state was assigned according to their read depth respect to the flanking regions (Additional file 1: Table S1).

The CNVRs were then identified using the 930 remaining consensus_CNVs. Each of the 389 identified CNVR (including 269 singletons) was visually validated to identify false positive calls, including all detected singletons. An example for the CNVR validation is shown in Additional file 2: Figure S1 at chrD1:10,624,001-10,645,000 where the comparison with the reference genome and two normal state cats, ABY_1 and BEN_1, clearly allows to identify true CNV calls. A total of 154 CNVRs were, containing 589 validated consensus_CNVs, were confirmed. Among those, 62 were identified as singleton. Table 2 summarizes the statistics of consensus_CNVs found in each breed.

**Table 2:** Descriptive statistics of validated copy number variant (consensus_CNVs) identified for each breed

Breed	N. of samples	Tot N. of CNV (*)	Min N. of CNV per sample	Max N. of CNV per sample	Tot N. Losses	Tot N. gains	Tot N. of Chr with CNV
ABY	3	46 (15.3)	13	17	42	4	13
BEN	2	29 (14.5)	12	17	25	4	14
BIR	6	95 (15.8)	11	20	88	7	16
BSH	2	19 (9.5)	9	10	17	2	7
BUR	5	78 (15.6)	10	20	67	11	16
EGY	1	19	19	19	18	1	14
MCO	2	24 (12)	12	12	23	1	9
NAP	3	53 (17.6)	16	20	47	6	14
OSH	8	136 (17)	10	28	117	19	17
PER	1	11	11	11	10	1	7
RAG	2	17 (9)	4	14	16	1	9
REX	2	22 (11)	10	12	19	3	12
SIA	3	21 (7)	4	12	17	4	11
SPH	1	19	19	19	15	4	11
Total	41	589			521	68	

(*) average number of CNV per Breed

**Fig. 1:**
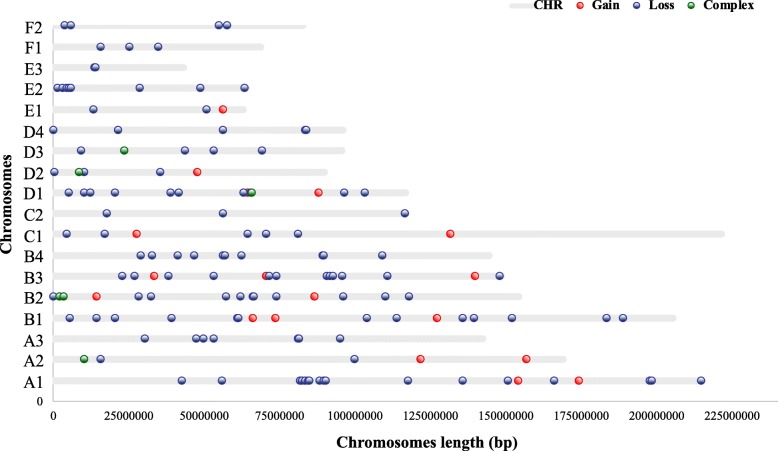
Physical distribution of the Copy Number Variants Regions (CNVRs) according to states (gain, loss and complex) on the *Felis catus* vs 6.2 assembly

The size of the singleton regions (62 CNVRs) ranged from 5 Kb to 283 Kb, while the remaining 92 CNVRs ranged in size from 5 Kb to 529 Kb. The 154 CNVRs and are graphically represented in Fig. 1 and reported in Additional file 3: Table S2 and Additional file 4: Table S3.

**Fig. 2:**
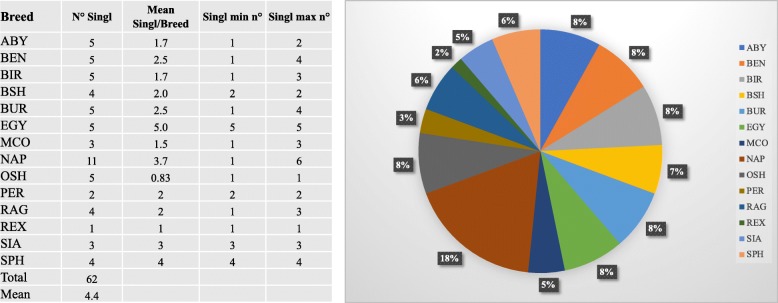
Summary of the number of singletons for each breed and breed contribution to the total number of detected singletons

Considering the length of the cat autosomes mapped with *Felix catus* vs 6.2 assembly (about 2.2 Gb), the CNVRs covered about 0.26% of the cat genome. A total of 129 loss, 19 gain and 6 complex regions were identified. The statistics and the contribution of each breed in relation to singletons are reported in Fig. 2. All breeds show to have at least one singleton CNVR with the NAP showing the largest number.

The contribution of every individual to each CNVR is reported in Additional file 4: Table S3. Two CNVRs were in common to the 66% of the cats: the first on chrD4:83,618,990-83,634,627, shared by 28 cats of 10 breeds and the second on chromosome chrA1:89,919,879-89,940,219, shared by 27 cats of 13 breeds. The CNVRs identified in at least 10 individuals were 13, while 26 CNVRS were present in only two cats. A total of 16 CNVRs were found in only one breed (ABY, BIR, BSH, BUR, MCO, NAP and OSH) as reported in Table 3.

**Table 3:** CNVRs found in only one breed

Chr	Start	End	Length	Breed	Samples (*)	State	Genes	Function
chrA1	117,723,730	117,738,524	14,794	ABY	2 (67)	Loss	PCDHB6, PCDHB10, PCDHB12	Neural cadherin-like cell adesion protein
chrB2	74,110,332	74,120,349	10,017	BIR	2 (25)	Loss	SH3BGRL2	SH3 Domain Binding Glutamate Rich Protein Like 2
chrD1	103,586,099	103,601,179	15,080	BIR	3 (60)	Loss	LOC101085660	Olfactory Receptor 5G3-like
chrD4	83,882,234	83,893,715	11,481	BIR	5 (83)	loss		
chrE1	56,306,407	56,332,265	25,858	BIR	3 (60)	Loss		
chrB2	96,292,366	96,391,191	98,825	BSH	2 (25)	Loss		
chrB3	70,859,943	71,389,394	529,451	BUR	2 (25)	Gain	AVEN	Apoptosis and Autophagy Pathways
							CHRM5	Muscarinic receptor
							EMC7	Membrane Protein binding carbohydrates
							RYR3	Ryanodine receptor for calcium release
chrB4	109,221,798	109,251,945	30,147	BUR	3 (60)	Loss		
chrE2	28,876,777	28,888,767	11,990	BUR	3 (60)	Loss		
chrA1	82,527,688	82,544,767	17,079	MCO	2 (100)	Loss		
chrC2	116,748,111	116,766,145	18,034	NAP	2 (25)	Loss	PLSCR4	Phospholipids migration by calcium ions binding
chrA2	121,965,379	121,983,429	18,050	OSH	2 (25)	Gain	ZNRF2	Maintenance of neural transmission
chrB1	139,721,970	139,739,225	17,255	OSH	2 (25)	Loss	CB1H4orf22	Cilia and flagella associated protein 299
chrB2	110,152,121	110,360,880	208,759	OSH	2 (25)	Loss		
chrB4	89,793,534	89,841,761	48,227	OSH	2 (25)	Loss	FAM19A2	TAFA family, regulators of immune and nervous cells
chrC2	17,974,294	17,988,686	14,392	OSH	2 (25)	Loss		

*Number of samples defining the CNVR and proportion on total number of cats per breed (%)

**Fig. 3:**
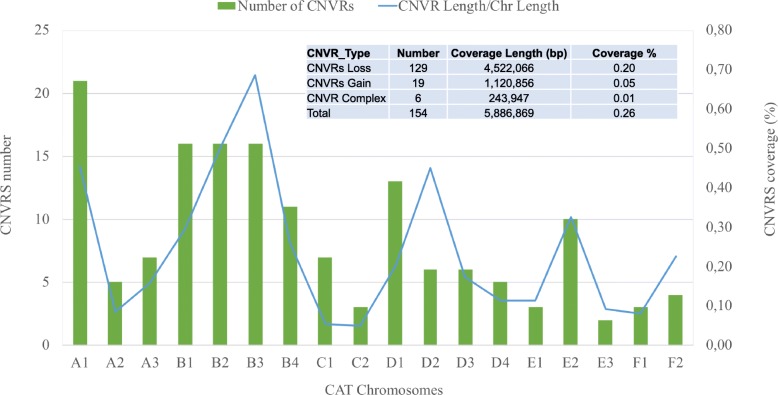
Distribution of CNVRs across the genome

In Fig. 3 the distribution of the CNVRs across the genome is reported together with the proportion of coverage within each chromosome. The number of CNVR per chromosome spans from 2 (chrE1) to 21 (chrA1) while the proportion of the total CNVR per chromosome spans from 0.05% (chrC2) to 0.69% (chrB3).

**Fig. 4:**
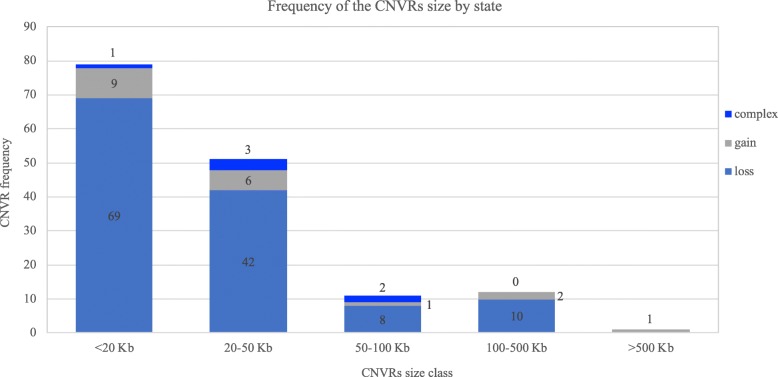
Distribution of CNVR size by class

In Fig. 4 the distribution of CNVRs by five size classes is reported. Only one CNVR, a gain, showed a size large than 500 Kb (chrB3:70,859,943-71,389,394, 520 Kb), while 67 are smaller than 20 Kb.

Considering the assembly_6.2 annotation, 46 genes were located within the CNVRs (Additional file 3: Table S2 and Additional file 4: Table S3) and 13 mapped in CNVRs identified in only one breed (Table 3).

All the 46 identified genes were submitted to the David Bioinformatic Database but for only 25 genes the GO terms and KEGG metabolic pathways were available as reported in Additional file 5: Table S4.

The GO terms and the KEGG pathway clusters, resulting from the DAVID classification database, are reported in Table 4. Only two clusters have a *P*-Value lower than 0.05 and correspond to a biological process term (G-protein coupled receptor signaling pathway) and a KEGG pathway (Olfactory transduction).

**Table 4:** Genes clusters according to DAVID database classification

Category	Term	Count	P-Value	Genes
GOTERM_BP	GO:0007186~G-protein coupled receptor signaling pathway	3	*9.14E-02*	LOC101101252, LOC101084174, LOC101083150
GOTERM_MF	GO:0005509~calcium ion binding	3	1.35E-01	MICU1, ANXA10, RYR3
GOTERM_MF	GO:0004984~olfactory receptor activity	3	1.27E-01	LOC101101252, LOC101084174, LOC101083150
GOTERM_MF	GO:0004930~G-protein coupled receptor activity	3	1.82E-01	LOC101101252, LOC101084174, LOC101083150
GOTERM_CC	GO:0016021~integral component of membrane	5	6.06E-01	ANTXRL, LOC101101252, RYR3, LOC101084174, LOC101083150
GOTERM_CC	GO:0005886~plasma membrane	3	6.28E-01	LOC101101252, LOC101084174, LOC101083150
GOTERM_CC	GO:0005737~cytoplasm	4	6.07E-01	ELP4, SYDE2, PAX6, ARNTL2
KEGG_PATHWAY	fca04740:Olfactory transduction	8	*1.10E-05*	LOC101095519, LOC101089503, LOC101101252, LOC101084174, LOC101089105, LOC101083150, LOC101083405, LOC101086964

*CC cellular component, *MF* molecular function

### Cat population analyses

**Fig. 5:**
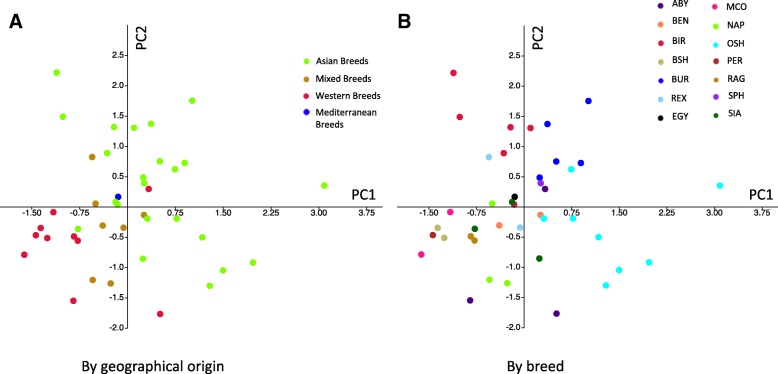
Scatter plot showing groups of stratification

**Fig. 6:**
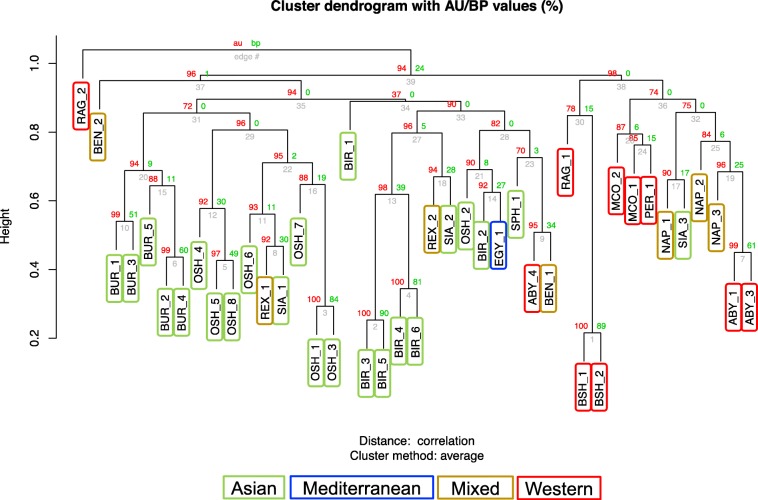
Clustering analysis using CNVRs. Colored rectangles correspond to geographical origin

Both the PCA and the cluster analysis depicted a similar population stratification based on different breeds (Figs. 5, 6).

Figure 5 shows the population stratification based on geographical origin (A) and on breeds (B). The principal components explained the 9.62 and 8.63% of the variance for PC1 and PC2 respectively. The breeds were grouped according to their geographical origin as follows: i) Asian Breeds - SIA, OSH, BUR, BIR and SPH; ii) Western Breeds - ABY, MCO, BSH, PER and RAG; iii) Mediterranean breed - EGY; iv) Mixed breeds – BEN, REX and NAP.

The clustering in Fig. 5-a shows two main groups of individuals represented by Asian breeds (green) and Western breeds (red). In Fig. 5-b a clear separate clustering is shown for BIR, BUR and OSH that are part of the Asian breeds group in Fig. 5-a. Among the Western breeds the BSH the PER and the RAG cluster very closely (Fig. 5-a).

The CNVR clustering tree also presented close associations for individuals belonging the same breed and for breeds with similar origins (Fig. 6).

The breeds clustered well according to the geographical origin, except for very few cases (RAG_2 and ABY_4 for the Western breeds; SIA_3 for the Asian breeds). For breeds with only one representative, the distribution was based on their geographical origins: Persian (PER_1) within the Western breed group while Egyptian (EGY_1) and Sphynx (SPH_1) within the Eastern breed group. Additionally, cats pertaining to the same breeds tend to cluster together as, e.g. the Burmese cats. Clustering with AU-*P* values > 90 were identified for several groupings of breeds, as for groups of individuals sharing the same geographical origins.

## Discussion

Genome-wide mapping of CNVs has allowed new insights into genomic variation. Different techniques based on different approaches have been developed with the aim of more efficient and accurate CNV detection [28]. While aCGH has been used to detect CNVs for several years, the advent of NGS technologies has improved the global detection and analysis of CNV data. In humans, as in domestic animals, different CNV analyses have already been performed and have demonstrated the strong relationship between the presence of CNVs and phenotypic diversity, evolution and the onset of several diseases [33, 12]. Although the examination of repetitive elements was carried out in cats [25] and a preliminary annotation of the whole genome reference sequence (*Felis catus* assembly_6.2) has revealed that repetitive elements comprise about 55.7% of the whole genome, no formal studies focusing on CNVs have been performed [34].

This is the first CNV detection in the domestic cat and also the first one using NGS data. CNVnator and cn.MOPS software were selected to identify the CNVs. Both packages are based on the Read Depth (RD) method, which has been suggested as the most suitable for Illumina sequencing data [28]. Compared to other methods, the RD method does not require a reference sample and is based on coverage of the sequencing data. This represents an important advantage as, during the detection, the software compares reads within the same sample and also among all the samples. Moreover, the RD method uses a negative binomial distribution statistical model [28] and has the advantage of setting the WL depending on the number of reads. The difference between the two software programs is based on the RD method pipelines described by [35]. Cn.MOPS is based on a Bayesian approach that measures the depth of coverage at each genomic position, across multiple samples, thus increasing the statistical power and decreasing the false discovery rate in the detection. CNVs and noise identification are achieved using mixture components and Poisson distribution [26]. In contrast, CNVnator uses the mean-shift approach [36] and CNV detection is made more accurate through GC and multiple-bandwidth partitioning corrections [35]. The significant difference in the number of detected CNVs between the two packages is based on the different approach for defining a single CNV. In cn.MOPS, copy number of adjacent windows are compared and those with the same copy number sequence are joined into one single CNV. The length of the final CNV is given by the sum of the two adjacent copy number sequences. The more adjacent segments with high or low copy number call are joined, the higher will be the confidence in the detections. CNVnator does not use this overlapping approach, which explains the large discrepancy in the number of CNVs detected by the two packages. Ten or more CNVs detected with CNVnator correspond to one CNV detected with cn.MOPS. For this reason, it was decided to obtain CNVRs after the selection of CNVs in common to the two software.

The number of analyzed individuals is comparable or superior to those used in previous studies on horses (16 individuals [37]), pigs (20 individuals [38]) and dogs (34 individuals [39]). The number and the mean lengths of CNVs are in accordance to those found in dogs, where 1748 CNVs with a mean length of 194,559 bp were identified [40], and in other studies using cn.MOPS [41] and CNVnator software [17].

The present CNV detection revealed a higher number of loss regions compared to gain and complex regions. This could be explained by the difference within breeds of the same species, as already reported for Holstein cattle, where the number of loss regions is consistently higher than in other cattle breeds [42, 43]. Moreover, even though CNVnator is still the most used software for CNV detection, it is known to have a major deficiency in terms of detecting gain regions [27]. This could also explain the identification of 147 CNVs with different state when the output of the two software packages was compared. Nevertheless, the visual inspection approach used in this study to validate all the CNVRs, allows to overcome the problem of having different calling state between the cn.MOPS and CNVnator software. Additionally, it further reduces the false positive calls that may occur when using only one of the software for the CNV detection. In fact, the visual inspection leads to a full validation of the called CNV, improving the true calls obtained by comparing the results of the two calling algorithms. Visual inspection was also used to reassign the boundaries at each single CNVR, allowing to further refine the genome proportion covered by CNVR. The singletons detected in this study represent the 41% of the total CNVRs, a lower proportion compared to those reported in other studies [44–46].

The proportion of validated non singleton CNVRs after visual inspection was 77%. This proportion of true calls is in accordance to the findings of [47] who identified a concordance for the 80% of mapped CNV using two sequencing data runs.

Grouping the individuals according to their CNVR similarities, both the PCA and the clustering analyses showed cats belonging to the same breed tended to cluster together as well as cats sharing the same geographical origin. The Western and Eastern breeds were distinct in both of the analyses and resembled the results obtained in previous studies of cat phylogenies [20, 48]. However, the Devon Rex cats, a breed developed in the United Kingdom [49], neither cluster nor have apparent correct historical origins, which are suggested as Eastern in this study. Depending on breeding associations, the genetic contributions of some cat breeds can be varied as different associations allow for different outcrosses for a given breed. Thus, some of the variation in the breed and individual associations may be due to historical breeding differences.

The Bengal breed is a hybrid, developed from crossing spotted cats from India, Egyptian Maus or Abyssinians with the Asian leopard cat (*Prionailurus bengalensis*). Thus, the convoluted genetics of an individual cat of the Bengal breed could easily result in placement nearly anywhere within the cat genetic spectrum. As previously stated [50], the Abyssinian, which is one of the oldest cat breeds, has mysterious origins that are not clearly defined.

The gene annotation performed in this study showed that 19% of the CNVRs harbor genes. This proportion is lower than the ones found in studies developed in other species [8, 40, 46]. This is likely due to the more incomplete information in the cat gene annotation, compared to other species.

The *LOC101085660, LOC101095519*, *LOC101101252* and *LOC101089105* genes encode for olfactory receptors and were found in CNVRs of several breeds. As previously shown in humans [51], it may be related to significant variability in olfactory capabilities, an important sensorial attribute in predators.

The *RYR3* gene in the Burmese cat was found differentially expressed in adipose tissue in cats during winter/short days and summer/long days [52].

The *SYDE2, PAX6, ELP4 and the CHRM5* genes, have been found in genomic regions that have been recognized to be under positive selection in cats during their domestication [53]. Interestingly, the *PAX6* and the *ELP4* genes were found by the same authors as genes underlying segmental duplication in the domestic cat genome, as occurring in this study.

The *PTCHD3* gene was found in the CNVR (complex) at chrB2:2,291,441-2,347,122 in 5 breeds (BEN, BIR, OSH, RAG, SPH). This gene has been previously found in a rare CNV in humans [54] where the CNV homozygous deletion was not associated to an abnormal phenotype. More recently, [55] have found this gene as associated with diabetes in humans and [56] have classified the gene as a potential imprinted gene. The *PTCHD3* gene is not yet included in the Imprinted Genes database (www.geneimprint.com), as no other gene found in the CNVRs here reported. The presence of imprinted genes in CNVRs has already been shown in other species such as in cattle [57]. The regulation of the gene expression is mainly determined by the genetic imprinting and it could be interesting to further investigate this aspect in cat too.

## Conclusions

The CNV calling performed in this study represents the first effort for the detection of genomic structural variation in the domestic cat. The clustering among the cat breeds that was possible to obtain in this study using CNVRs, complement findings of other studies based on other type of markers, leading to a closer insight of common and proprietary functional aspects of each population. Further studies based on further resequencing and on novel NGS technologies, might disclose other insights on CNV in *Felis catus* species and could complement the results obtained with the mapping performed in this study. Since CNV are well known to be related to gene expression regulation, also in complex diseases, this first mapping is meant to be the first information on a class of genomic variants that can be related to recorded phenotypes in cat populations.

## Additional files

Additional file 1: **Table S1.** (XLSX). Consensus CNVs. List of the consensus list of CNVs detected by both cn.mops and CNVnator software and state confirmed with visual inspection with GenomeBrowse (XLSX 34 kb)

Additional file 2: **Figures S1.** (PDF). Visual inspection of CNVR at chrD1:10624094–10,643,050. (TIF 2999 kb)

Additional file 3: **Table S2.** (XLSX). Singleton CNVRs and list of annotated genes. (XLSX 14 kb)

Additional file 4: **Table S3.** (XLSX). List of CNVRs. List of the CNVRs and annotated genes. Singletons were excluded. (XLSX 20 kb)

Additional file 5: **Table S4.** (DOCX). David annotation of genes in all CNVRs. List of the annotation gene names GO terms (BP: biological process; CC: cellular component; MF: molecular function), and KEGG pathways. (DOCX 22 kb)

## Abbreviations

ABY: Abyssinian breed
aCGH: Array-Based Comparative Genomic Hybridization
AU-P: Approximately Unbiased *P*-value
BEN: Bengal breed
BIR: Birman breed
BP-P: Bootstrap Probability value
BSH: British Short Hair breed
BUR: Burmese breed
CNV: Copy Number Variants
CNVR: Copy Number Variants Region
EGY: Egyptian breed
GO: Gene Ontology Terms
KEGG: Kyoto Encyclopedia of Genes and Genomes
LCVs: Large-Scale Copy-Number Variants
MCO: Main Coon breed
NAP: Napoleon breed
NGS: Next Generation Sequencing
OSH: Oriental Short Hair breed
PER: Persian breed
RAG: Ragdoll breed
RD: Read Depth
REX: Devon Rex breed
SIA: Siamese breed
SNP: Single Nucleotide Polymorphism
SPH: Sphynx breed
UPGMA: Unweighted Pair-Group Average
