# Supplementary figures and images for: First genome-wide CNV mapping in FELIS CATUS using next generation sequencing data
Source: BMC Genomics. 2018 Dec 10;19:895. doi: 10.1186/s12864-018-5297-2 (PMC6288940; doi:10.1186/s12864-018-5297-2)

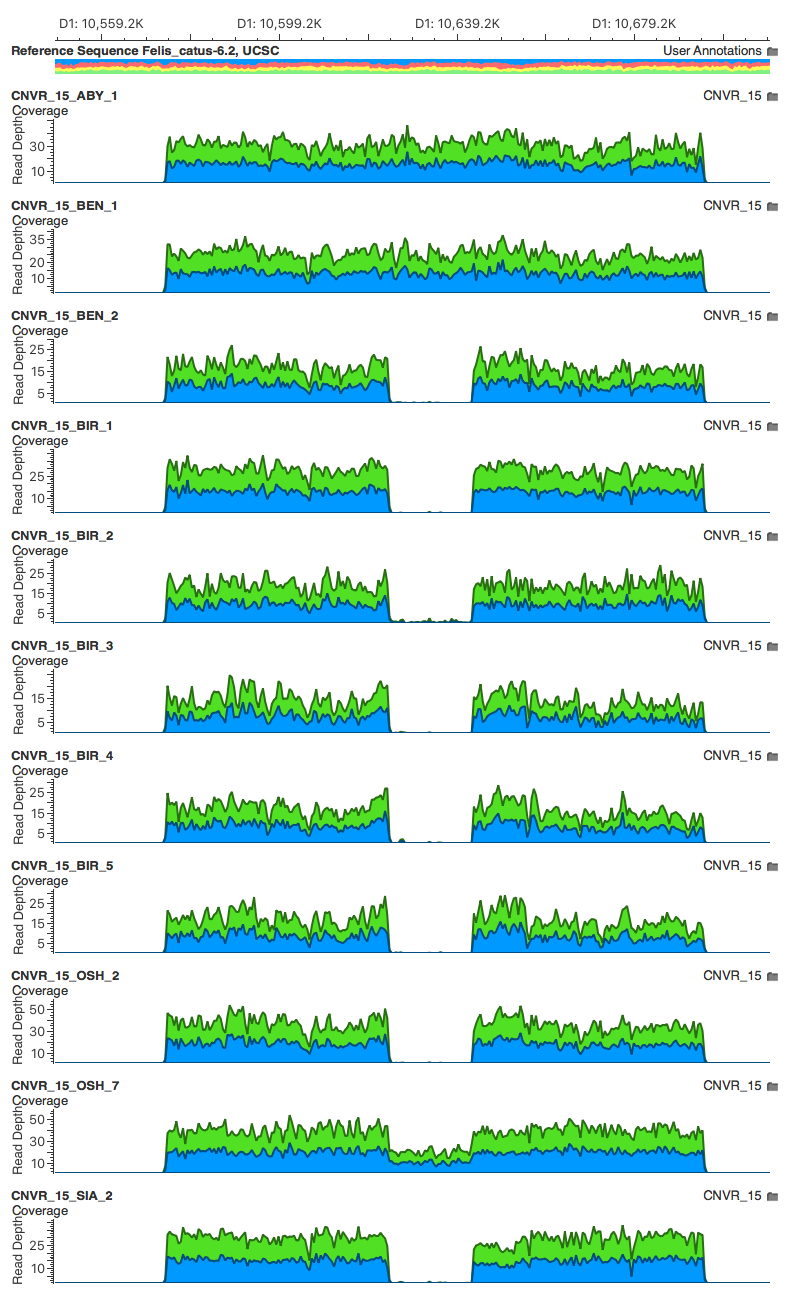

Supplement: Supplementary file 2 — Figures S1. (PDF). Visual inspection of CNVR at chrD1:10624094–10,643,050. (TIF 2999 kb) [file 12864_2018_5297_MOESM2_ESM.tif]
