# Supplementary material for: First genome-wide CNV mapping in FELIS CATUS using next generation sequencing data
Source: BMC Genomics. 2018 Dec 10;19:895. doi: 10.1186/s12864-018-5297-2 (PMC6288940; doi:10.1186/s12864-018-5297-2)
Supplement: Supplementary file 5 — Table S4. (DOCX). David annotation of genes in all CNVRs. List of the annotation gene names GO terms (BP: biological process; CC: cellular component; MF: molecular function), and KEGG pathways. (DOCX 22 kb) [file 12864_2018_5297_MOESM5_ESM.docx]

**Table S4**. **David annotation of genes included in the all CNVRs.** List of the annotation gene names GO terms (BP: biological process; CC: cellular component; MF: molecular function), and KEGG pathways.

| **ID** | **Gene Name** | **GOTERM_BP_DIRECT** | **GOTERM_CC_DIRECT** | **GOTERM_MF_DIRECT** | **KEGG_PATHWAY** |
| --- | --- | --- | --- | --- | --- |
| **EMC7** | ER membrane protein complex subunit 7 |  | GO:0072546~ER membrane protein complex | GO:0030246~carbohydrate binding |  |
| **SH3BGRL2** | SH3 domain binding glutamate rich protein like 2 |  | GO:0005654~nucleoplasm, GO:0070062~extracellular exosome |  |  |
| **THOC3** | THO complex 3 | GO:0006406~mRNA export from nucleus, GO:0046784~viral mRNA export from host cell nucleus | GO:0000445~THO complex part of transcription export complex, GO:0000784~nuclear chromosome, telomeric region |  | fca03013:RNA transport, fca03040:Spliceosome |
| **ANXA10** | annexin A10 |  | GO:0005739~mitochondrion | GO:0005509~calcium ion binding, GO:0005544~calcium-dependent phospholipid binding |  |
| **ANTXRL** | anthrax toxin receptor-like |  | GO:0016021~integral component of membrane | GO:0004872~receptor activity |  |
| **AVEN** | apoptosis and caspase activation inhibitor | GO:0043066~negative regulation of apoptotic process | GO:0005622~intracellular,  GO:0016020~membrane |  |  |
| **ARNTL2** | aryl hydrocarbon receptor nuclear translocator like 2 | GO:0006351~transcription, DNA-templated, GO:0007623~circadian rhythm, GO:0042753~positive regulation of circadian rhythm, GO:0045944~positive regulation of transcription from RNA polymerase II promoter | GO:0005667~transcription factor complex, GO:0005730~nucleolus, GO:0005737~cytoplasm | GO:0000982~transcription factor activity, RNA polymerase II core promoter proximal region sequence-specific binding, GO:0070888~E-box binding |  |
| **CHRM5** | cholinergic receptor muscarinic 5 | GO:0001696~gastric acid secretion,  GO:0007197~adenylate cyclase-inhibiting G-protein coupled acetylcholine receptor signaling pathway,  GO:0007207~phospholipase C-activating G-protein coupled acetylcholine receptor signaling pathway,  GO:0007271~synaptic transmission, cholinergic,  GO:0015872~dopamine transport,  GO:0019226~transmission of nerve impulse | GO:0005887~integral component of plasma membrane, GO:0030054~cell junction, GO:0045202~synapse, GO:0045211~postsynaptic membrane | GO:0016907~G-protein coupled acetylcholine receptor activity | fca04020:Calcium signaling pathway, fca04080:Neuroactive ligand-receptor interaction, fca04725:Cholinergic synapse,  fca04810:Regulation of actin cytoskeleton |
| **ELP4** | elongator acetyltransferase complex subunit 4 | GO:0006357~regulation of transcription from RNA polymerase II promoter,  GO:0006368~transcription elongation from RNA polymerase II promoter,  GO:0043966~histone H3 acetylation,  GO:0043967~histone H4 acetylation | GO:0000123~histone acetyltransferase complex,  GO:0005737~cytoplasm,  GO:0008023~transcription elongation factor complex,  GO:0033588~Elongator holoenzyme complex | GO:0000993~RNA polymerase II core binding |  |
| **MICU1** | mitochondrial calcium uptake 1 | GO:0036444~calcium ion transmembrane import into mitochondrion,  GO:0051260~protein homooligomerization,  GO:0051561~positive regulation of mitochondrial calcium ion concentration | GO:0005758~mitochondrial intermembrane space,  GO:0032592~integral component of mitochondrial membrane,  GO:1990246~uniplex complex | GO:0005509~calcium ion binding |  |
| **LOC101095519** | olfactory receptor 10A2 |  |  |  | fca04740:Olfactory transduction |
| **LOC101101252** | olfactory receptor 10A5 | GO:0007186~G-protein coupled receptor signaling pathway,  GO:0007608~sensory perception of smell, | GO:0005886~plasma membrane,  GO:0016021~integral component of membrane | GO:0004930~G-protein coupled receptor activity, GO:0004984~olfactory receptor activity | fca04740:Olfactory transduction |
| **LOC101083405** | olfactory receptor 7A17-like |  |  |  | fca04740:Olfactory transduction |
| **LOC101086964** | olfactory receptor 9A4-like |  |  |  | fca04740:Olfactory transduction |
| **LOC101083150** | olfactory receptor-like protein OLF4 | GO:0007186~G-protein coupled receptor signaling pathway,  GO:0050911~detection of chemical stimulus involved in sensory perception of smell | GO:0005886~plasma membrane,  GO:0016021~integral component of membrane | GO:0004930~G-protein coupled receptor activity,  GO:0004984~olfactory receptor activity | fca04740:Olfactory transduction |
| **LOC101084174** | olfactory receptor-like protein OLF4 | GO:0007186~G-protein coupled receptor signaling pathway,  GO:0050911~detection of chemical stimulus involved in sensory perception of smell | GO:0005886~plasma membrane,  GO:0016021~integral component of membrane | GO:0004930~G-protein coupled receptor activity,  GO:0004984~olfactory receptor activity | fca04740:Olfactory transduction |
| **PAX6** | paired box 6 | GO:0000132~establishment of mitotic spindle orientation,  GO:0001568~blood vessel development,  GO:0001709~cell fate determination,  GO:0001764~neuron migration,  GO:0001933~negative regulation of protein phosphorylation,  GO:0002052~positive regulation of neuroblast proliferation,  GO:0002088~lens development in camera-type eye,  GO:0003309~type B pancreatic cell differentiation,  GO:0003322~pancreatic A cell development,  GO:0007224~smoothened signaling pathway,  GO:0007411~axon guidance,  GO:0007435~salivary gland morphogenesis,  GO:0009611~response to wounding,  GO:0009786~regulation of asymmetric cell division,  GO:0009950~dorsal/ventral axis specification,  GO:0021778~oligodendrocyte cell fate specification,  GO:0021796~cerebral cortex regionalization,  GO:0021798~forebrain dorsal/ventral pattern formation,  GO:0021902~commitment of neuronal cell to specific neuron type in forebrain,  GO:0021905~forebrain-midbrain boundary formation,  GO:0021912~regulation of transcription from RNA polymerase II promoter involved in spinal cord motor neuron fate specification,  GO:0021913~regulation of transcription from RNA polymerase II promoter involved in ventral spinal cord interneuron specification,  GO:0021918~regulation of transcription from RNA polymerase II promoter involved in somatic motor neuron fate commitment,  GO:0021983~pituitary gland development,  GO:0021986~habenula development,  GO:0023019~signal transduction involved in regulation of gene expression,  GO:0030216~keratinocyte differentiation,  GO:0030334~regulation of cell migration,  GO:0030858~positive regulation of epithelial cell differentiation,  GO:0032808~lacrimal gland development,  GO:0033365~protein localization to organelle,  GO:0042462~eye photoreceptor cell development,  GO:0042593~glucose homeostasis,  GO:0045665~negative regulation of neuron differentiation,  GO:0048505~regulation of timing of cell differentiation,  GO:0048596~embryonic camera-type eye morphogenesis,  GO:0048708~astrocyte differentiation,  GO:0050680~negative regulation of epithelial cell proliferation,  GO:0060041~retina development in camera-type eye,  GO:0061072~iris morphogenesis,  GO:0061303~cornea development in camera-type eye,  GO:2000178~negative regulation of neural precursor cell proliferation, | GO:0000790~nuclear chromatin,GO:0005654~nucleoplasm,GO:0005737~cytoplasm, | GO:0000978~RNA polymerase II core promoter proximal region sequence-specific DNA binding, GO:0000979~RNA polymerase II core promoter sequence-specific DNA binding, GO:0001077~transcriptional activator activity, RNA polymerase II core promoter proximal region sequence-specific binding, GO:0001227~transcriptional repressor activity, RNA polymerase II transcription regulatory region sequence-specific binding, GO:0003682~chromatin binding |  |
| **KCNH5** | potassium voltage-gated channel subfamily H member 5 | GO:0010389~regulation of G2/M transition of mitotic cell cycle,  GO:0042391~regulation of membrane potential | GO:0005622~intracellular,  GO:0005887~integral component of plasma membrane,  GO:0009986~cell surface | GO:0000155~phosphorelay sensor kinase activity,  GO:0005249~voltage-gated potassium channel activity, |  |
| **LOC101089105** | putative olfactory receptor 2B8 |  |  |  | fca04740:Olfactory transduction, |
| **LOC101089503** | putative olfactory receptor 2B8 |  |  |  | fca04740:Olfactory transduction, |
| **PYROXD1** | pyridine nucleotide-disulphide oxidoreductase domain 1 |  |  | GO:0016491~oxidoreductase activity, |  |
| **RYR3** | ryanodine receptor 3 | GO:0051209~release of sequestered calcium ion into cytosol, | GO:0005623~cell,  GO:0005790~smooth endoplasmic reticulum,  GO:0016021~integral component of membrane,  GO:0030018~Z disc,  GO:0033017~sarcoplasmic reticulum membrane,  GO:0034704~calcium channel complex,  GO:0042383~sarcolemma | GO:0005219~ryanodine-sensitive calcium-release channel activity,  GO:0005509~calcium ion binding,GO:0048763~calcium-induced calcium release activity, | fca04020:Calcium signaling pathway,f  ca04713:Circadian entrainment,  fca04921:Oxytocin signaling pathway,  fca04970:Salivary secretion,  fca05010:Alzheimer's disease |
| **SNX25** | sorting nexin 25 | GO:0030512~negative regulation of transforming growth factor beta receptor signaling pathway,  GO:0032801~receptor catabolic process,  GO:0060394~negative regulation of pathway-restricted SMAD protein phosphorylation | GO:0005768~endosome | GO:0035091~phosphatidylinositol binding, |  |
| **SYDE2** | synapse defective Rho GTPase homolog 2 | GO:0007165~signal transduction,  GO:0043087~regulation of GTPase activity | GO:0005737~cytoplasm |  |  |
